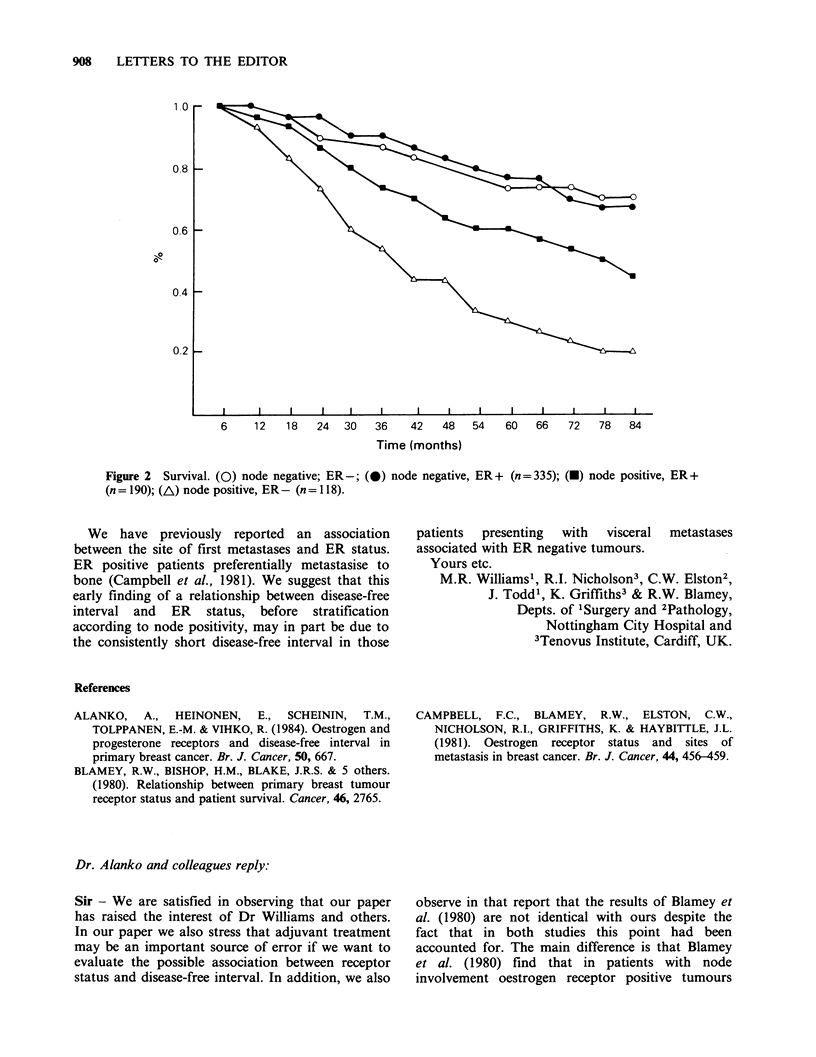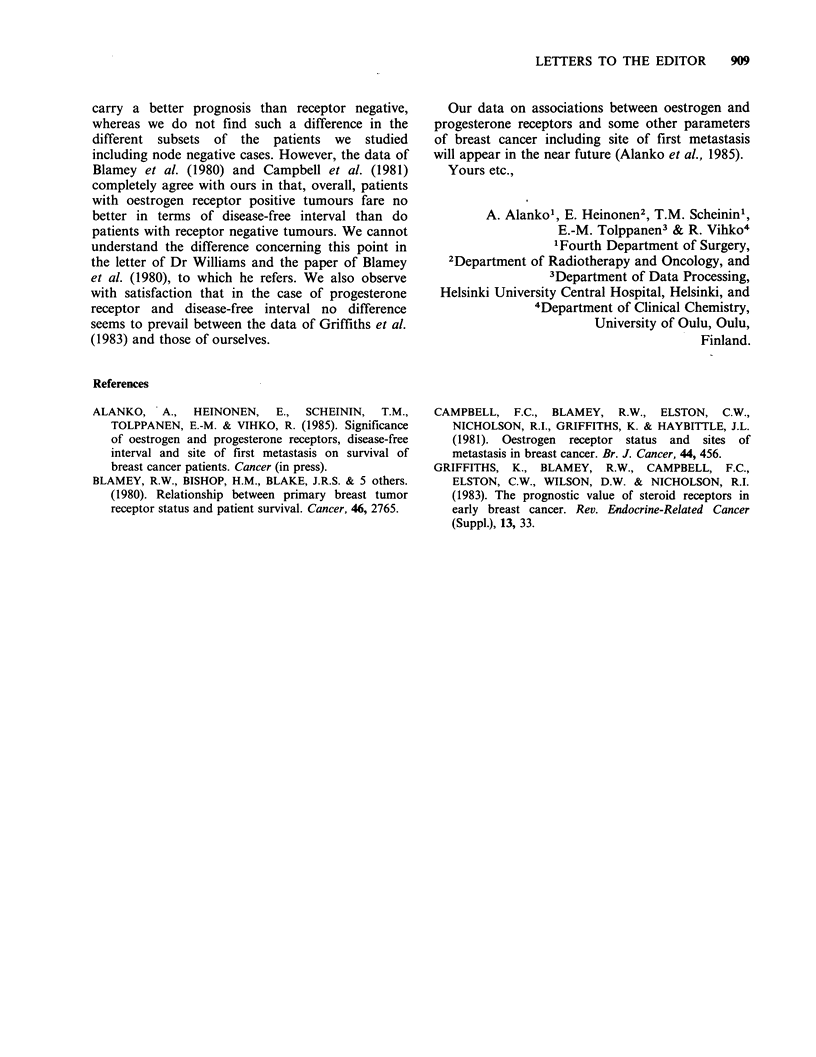# Oestrogen receptors in primary breast cancer

**Published:** 1985-06

**Authors:** A. Alanko, E. Heinonen, T.M. Scheinin, E.-M. Tolppanen, R. Vihko


*Dr. Alanko and colleagues reply:*

**Sir** – We are satisfied in observing that our paper has raised the interest of Dr Williams and others. In our paper we also stress that adjuvant treatment may be an important source of error if we want to evaluate the possible association between receptor status and disease-free interval. In addition, we also observe in that report that the results of Blamey *et al.* (1980) are not identical with ours despite the fact that in both studies this point had been accounted for. The main difference is that Blamey *et al.* (1980) find that in patients with node involvement oestrogen receptor positive tumours carry a better prognosis than receptor negative, whereas we do not find such a difference in the different subsets of the patients we studied including node negative cases. However, the data of Blamey *et al.* (1980) and Campbell *et al.* (1981) completely agree with ours in that, overall, patients with oestrogen receptor positive tumours fare no better in terms of disease-free interval than do patients with receptor negative tumours. We cannot understand the difference concerning this point in the letter of Dr Williams and the paper of Blamey *et al.* (1980), to which he refers. We also observe with satisfaction that in the case of progesterone receptor and disease-free interval no difference seems to prevail between the data of Griffiths *et al.* (1983) and those of ourselves.

Our data on associations between oestrogen and progesterone receptors and some other parameters of breast cancer including site of first metastasis will appear in the near future (Alanko *et al.*, 1985).

Yours etc.,

A. Alanko[1], E. Heinonen[2], T.M. Scheinin[1],
E.-M. Tolppanen[3] & R. Vihko[4]
[1]Fourth Department of Surgery,
[2]Department of Radiotherapy and Oncology, and
[3]Department of Data Processing,
Helsinki University Central Hospital, Helsinki, and
[4]Department of Clinical Chemistry,
University of Oulu, Oulu,
Finland.

**References**

ALANKO, A., HEINONEN, E., SCHEININ, T.M., TOLPPANEN, E.-M. & VIHKO, R. (1985). Significance of oestrogen and progesterone receptors, disease-free interval and site of first metastasis on survival of breast cancer patients. *Cancer* (in press).

BLAMEY, R.W., BISHOP, H.M., BLAKE, J.R.S. & 5 others. (1980). Relationship between primary breast tumor receptor status and patient survival. *Cancer*, **46**, 2765.

CAMPBELL, F.C., BLAMEY, R.W., ELSTON, C.W., NICHOLSON, R.I., GRIFFITHS, K. & HAYBITTLE, J.L. (1981). Oestrogen receptor status and sites of metastasis in breast cancer. *Br. J. Cancer*, **44**, 456.

GRIFFITHS, K., BLAMEY, R.W., CAMPBELL, F.C., ELSTON, C.W., WILSON, D.W. & NICHOLSON, R.I. (1983). The prognostic value of steroid receptors in early breast cancer. *Rev. Endocrine-Related Cancer* (Suppl.), **13**, 33.